# Implementing a digital communication assistance tool to collect the medical history of refugee patients: DICTUM Friedland - an action-oriented mixed methods study protocol

**DOI:** 10.1186/s12913-019-3928-1

**Published:** 2019-02-06

**Authors:** Ghefar Furaijat, Evelyn Kleinert, Anne Simmenroth, Frank Müller

**Affiliations:** 1 Department of General Practice, University Medical Centre Göttingen/Georg-August-University, Humboldtallee 38, 37073 Göttingen, Germany; 2 Department of General Practice, University Medical Centre Würzburg/Julius-Maximilian-University, Josef-Schneider-Straße 2, 97080 Würzburg, Germany

**Keywords:** Medical history, Digital, Interpretation, Primary healthcare, Refugees and asylum seekers

## Abstract

**Background:**

Language barriers play a decisive role in determining the outcomes of medical consultations between healthcare providers and their foreign patients. This issue is a significant challenge to the German healthcare system, especially with the rising number of refugees in recent years. The communication gap between healthcare professionals and their non-German speaking patients sometimes leads to unnecessary medical re-admission, insufficient medical history, incorrect diagnosis, and treatment plans. In this study, we aim to assess the usability and accuracy of a novel digital translation tool in collecting medical history from patients in their native language and to check its effects on healthcare outcomes.

**Methods:**

The study aims to monitor the implementation of a new digital communication assistance tool (DCAT) and to investigate its impact on the mutual understanding between refugee patients and their German general practitioners (GPs). In the first study phase, an action-oriented approach is used to implement DCAT. In the second study phase, DCAT use will be evaluated with a mixed methods design.

The main outcome assesses the re-consultation rates of patients before and after using DCAT. Secondary outcomes include the usability of the tool, its acceptance and perceived quality by patients, the accuracy of the information collected as determined from analysing the reasons for the consultation (ICPC-2 codes), and diagnosis (ICD-10 codes). The acceptance by patients, socio-demographic factors and native language are also taken into account.

The research designs for both study phases include questionnaires, semi-structured interviews, non-participant observation and analysis of collected patients’ data.

All the collected data is pseudonymised.

**Discussion:**

The DCAT study is one of the new research projects in primary healthcare investigating the usability, accuracy, and acceptance of digital translation tools during medical encounters. We aim to eliminate significant communication errors and misunderstandings in medical consultations, thereby improving the quality of healthcare outcomes. By applying an action research design, we will attain a more comprehensive evaluation of DCAT scopes and limits. The results of this study are expected to give an in-depth understanding of possible applications and benefits of digital translation tools for patient care.

**Trial registration:**

German Clinical Trials Register DRKS00013076, 29/09/2017.

**Electronic supplementary material:**

The online version of this article (10.1186/s12913-019-3928-1) contains supplementary material, which is available to authorized users.

## Background

Over one million refugees and asylum seekers have been registered in Germany within the last three years [1]. The German healthcare system faces considerable difficulties in providing adequate and high-quality healthcare for such a large number of people [2, 3]. Many factors influence the provision of healthcare to this group of patients, and some of the most important arise from language and cultural barriers [4]. These problems also contribute to the lack of patient knowledge of the German healthcare system [5, 6].

In most cases, healthcare professionals cannot rely on the accuracy of the medical history collected from refugees and asylum seekers due to the limited access to professional interpreters [7]. This also increases the risks of medical errors and misdiagnosis received by these patients as well as potential drug-related adverse effects and medical complications [8, 9]. While professional interpreters are usually not available for such a large number of patients, especially in rural areas [6], health policymakers generally prefer not to employ interpreter-services because of the financial burden this also places on the healthcare system [10] and the German public health insurance does not typically cover such expenses [11]. Additional concerns relating to the use of professional interpreters include privacy and patient-physician confidentiality as well as the interpretation quality for medical purposes [12].

The problem of language barriers in the German healthcare system has been appreciated for some time, but adequate practical solutions have not been forthcoming [2]. Digital approaches may bridge the communication gap during medical consultations and address the needs of both patients and healthcare providers correctly, especially when an interpreter is not available and there is a need for essential communication, i.e. in a spontaneous consultation.

There have been attempts to develop such digital tools. However, the quality and feasibility of these approaches have rarely been examined methodologically in a clinical context [13]. Whereas earlier tools focused mostly on self-diagnosis and treatment in the absence of healthcare professionals [8], this interventinon seeks to assist healthcare providers by collecting medical histories from the non-German speaking patients and then provide them with the collected relevant medical information in the German language. The primary goal of the DCAT is, therefore, to collect structured medical histories from non-German speaking patients in their native language and then to provide physicians with a corresponding German medical report to aid in patient primary care.

### The aim of the study

In this study, we explore DCATs usability, accuracy and the helpfulness as well as its impact on patient’s utilisation of medical services (re-consultations). By doing so, perspectives from all stakeholders (GPs, refugee patients and healthcare system insurers/providers) are taken into account.

The results of this study are expected to indicate the effectiveness of DCAT with respect to improving the communication with medical professionals and consequently medical outcomes for non-German speaking patients of the healthcare encounter. The study is located at a refugee camp in Lower Saxony in Germany. A daily GPs consultation hour at a primary healthcare centre in the camp is offered for acute medical conditions for all camp residents.

## Methods

### Design

The first study phase follows an action-oriented approach, meaning that observation outcomes can be used as an input to improve the content and implementation process of DCAT. This pragmatic approach allows us to make adjustments within a complex intervention in a barely known field, in response to unexpected problems and new opportunities that arise throughout the study. In contrast to other interventional procedures, the action research method requires close collaboration between researchers and other involved parties in the field [14, 15]. This approach also has proved its validity in evaluating health-related issues among refugees and asylum seekers [16, 17]. The “action” follows a circular process consisting of planning, implementation, observation and reflection, which is repeated several times. Since it is not possible to ascertain which challenges may be faced during the testing, it is difficult to determine ahead of time the duration of the action research method. This initial study phase ends with a successful three week preliminary testing with patients supported by intensive field observation and technical backup. Following this testing, the final version of DCAT will be fixed and will remain unchanged during the second study phase.

For a better understanding of DCAT impact, potential and limitations, a mixed methods research design is to be combined with quantitative and qualitative research methods in the second study phase [18, 19]. The quantitative approach includes DCAT-users and non-DCAT users and therefore offers the possibility to compare both groups in an interventional study design.

The primary outcome will be the consultation rate of patients who used DCAT compared with those who did not use this digital translation tool. As we found in a previous pilot study, the consultation rate of refugee patients at the primary healthcare centre is higher, especially compared to the non-refugee population, and this might be a result of miscommunication. Also, consultation rates are key values for healthcare costs.

As a secondary outcome, the following are to be assessed:

- The perceived quality of the communication in the consultation by both patients and healthcare professionals. After each consultation patients are asked to fill in questionnaires about their perceptions around mutual understanding with the attending physician.
- The perception of the usability and acceptance of the tool by patients and GPs, which is also gauged through questionnaires after each consultation.
- The usability and acceptance of DCAT by different patient subgroups according to age, gender, country of origin, asylum status, educational level and spoken languages. When patients decide to stop using DCAT, this will also be registered.
- The accuracy of collected information through DCAT. The aim is to clarify whether DCAT can be reliably utilised and incorporated into the clinical decision-making process to improve healthcare outcomes [20, 21]. DCAT estimates the reasons for the encounter as ICPC-2 codes based on the information provided by the patient, and doctors will also be indicating their diagnosis (ICD-10 codes). We want to compare the DCATs estimated reasons for a consultation and the GP’s diagnosis in terms of plausibility and contingency to investigate the accuracy of the provided medical information by DCAT [22]. Also, GPs will be asked after each consultation through questionnaires if their impression of the patient’s description contradicts the information provided by DCAT.

For a deeper insight into the acceptance of DCAT users, we will apply qualitative research methods, namely:

- Use of open and partially standardised interviews conducted with healthcare workers (GPs and nurses) and a group of selected patients in the refugee camp.
- The researchers also will explore the use of DCAT as a non-participant observer.

**Fig. 1:**
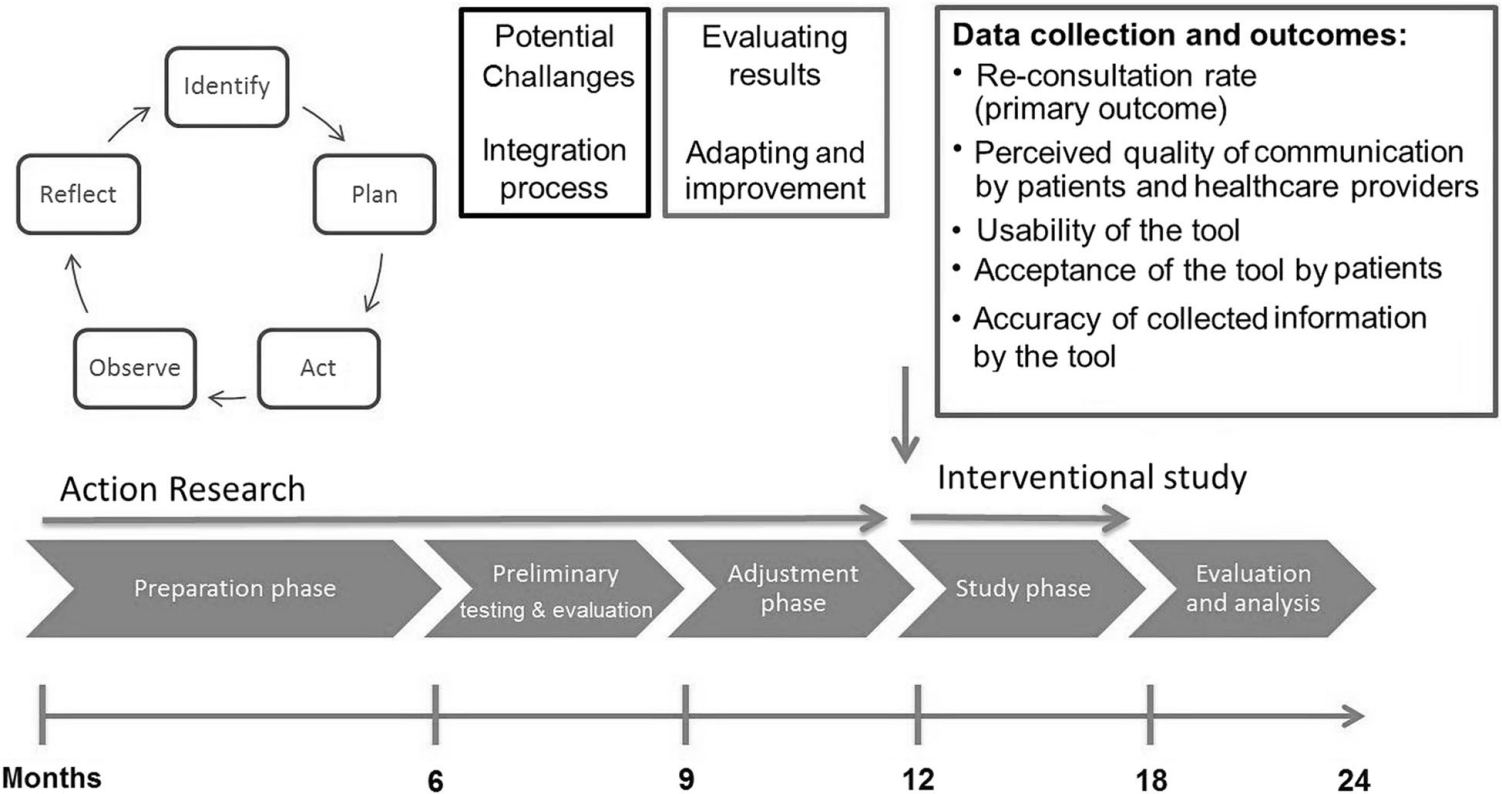
Research design and planned timeline of the DCAT study

The research design of the DCAT Study is shown in Fig. 1.

### Study setting

The study takes place in the Friedland transit camp “Grenzdurchgangslager Friedland”. The camp was established in 1945 in the middle of Germany, Lower Saxony. Since its foundation, it has received over 4 million people. Nowadays, it accommodates two groups of refugees: resettlement refugees (who have already gone through the refugee-status determination process) and asylum seekers (who have applied for asylum after arrival in Germany and are waiting for the decision regarding their refugee-status). Currently, there are 200–400 resettlement refugees and asylum seekers mainly from the middle east and north Africa countries [23]. The primary healthcare centre in Friedland is an outpatient clinic staffed by a team of six GPs offering medical consultations to refugees and asylum seekers during weekdays. These doctors work in co-operation with nursing staff from Malteser International, which also operates the facility outside regular consultation hours [24].

According to the Services for Asylum Seekers Act (AsylBLG sections 4 and 6) [2], only primary healthcare services for acute medical conditions are insured [11]. These conditions represent diseases and complaints commonly examined by primary healthcare doctors (medical emergencies, acute conditions, pregnancy and vaccinations) [25].

### The digital communication assistance tool (DCAT) concept

The digital communication assistance tool, which will be implemented and evaluated in the study, was developed by aidminutes GmbH, a former study group at Leuphana University in Lüneburg and the Department of General Practice at the Göttingen University Medical Centre.

**Fig. 2:**
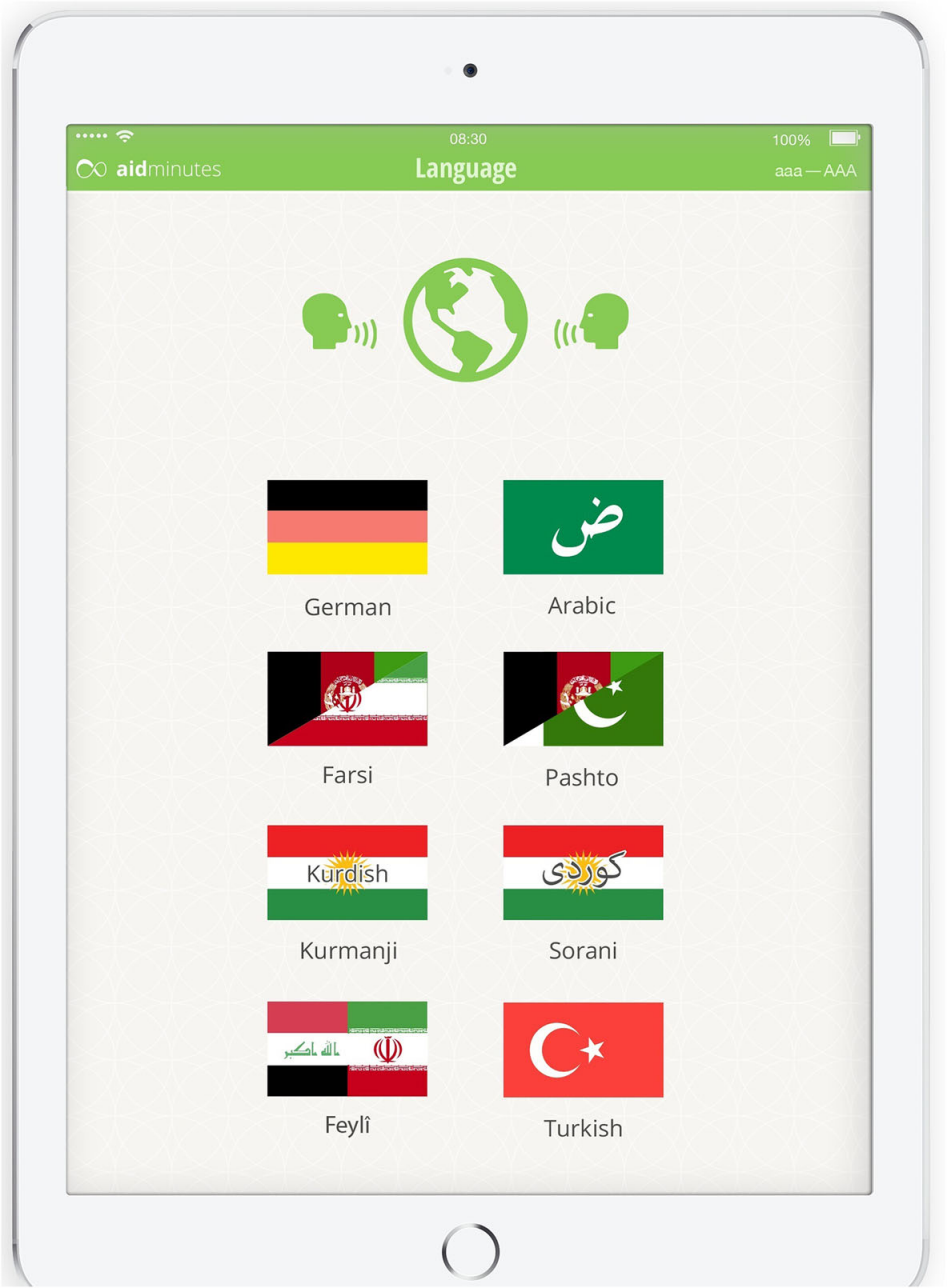
Available languages in the DCAT

**Fig. 3:**
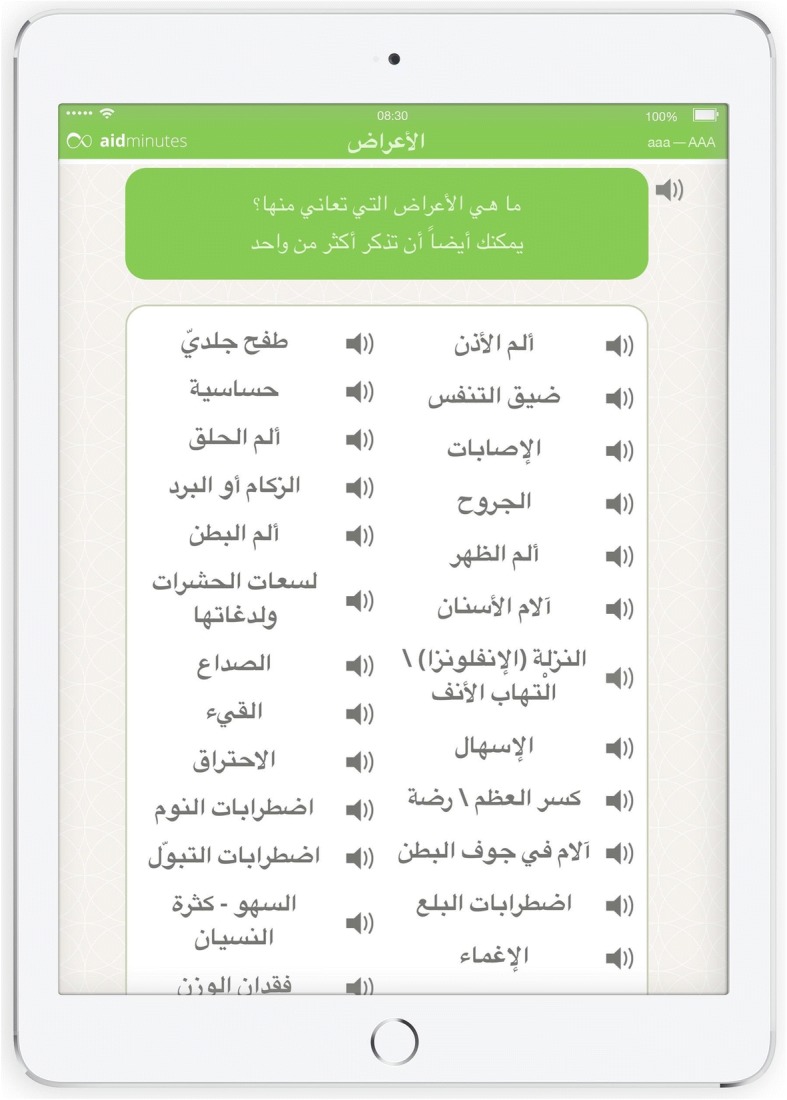
Main signs and symptoms window of DCAT displayed in Arabic

DCAT is able to collect medical histories in 13 different languages/dialects (Modern Standard Arabic, Arabic Syrian, Arabic Egyptian, Arabic Tunisian, Arabic Moroccan, Turkish, Persian, Kurdish Sorani, Kurdish Kurmanji, Kurdish Feyli, Pashto Kandahari, Pashto Mazurka) and generate a translated summary in German for the German-speaking doctors (see Figs. 2 and 3).

The questioning structures are partly based on the concept of “programmed diagnostics in general medicine” by Braun and Mader [14], a cybernetic approach for gaining information on common signs and symptoms as well as diagnostic procedures. Several GPs with significant experience in the medical care of migrants have developed the DCAT medical history algorithm and overseen the whole programming process. Professional interpreters translated the DCAT content in its entirety. Using DCAT, the patient is asked to specify a symptom (e.g. a headache) or concern (e.g. prescription of a medication). It is possible to include additional symptoms and order them by importance. At the same time, the symptoms input can be elaborated on through further inquiry and via the possibility of visual information.

### Study population

#### Participants and inclusion criteria

In this study, informed participants can use DCAT:

1. At the primary healthcare centre for patients seeking medical care and
2. At the social service bureau referral point.

All patients who meet the following inclusion criteria are eligible to participate in the study:

- Patients who speak one of the following languages: Arabic, Kurdish, Turkish, Persian, Pashto or any of the following dialects: Modern Standard Arabic (MSA), Arabic Syrian, Arabic Egyptian, Arabic Tunisian, Arabic Moroccan, Kurdish Sorani, Kurdish Kurmanji, Kurdish Feyli, Pashto Kandahari, Pashto Mazurka.
- The patients (or in case of children their parents) provide written informed consent before participating in the DCAT study.

### Recruitment and informed consent

Participating in the study and utilising DCAT is voluntary. Written informed consent is collected from each patient (or from the parents for patients under 18 years) willing to participate before using DCAT. All participants have the right to withdraw from the study at any point and all their collected information is immediately deleted.

Since the DCAT study is restricted to non-German-speaking patients, who might also have difficulties in reading and writing, audio- and visual content in the chosen language has been incorporated into DCAT [15, 16].

**Fig. 4:**
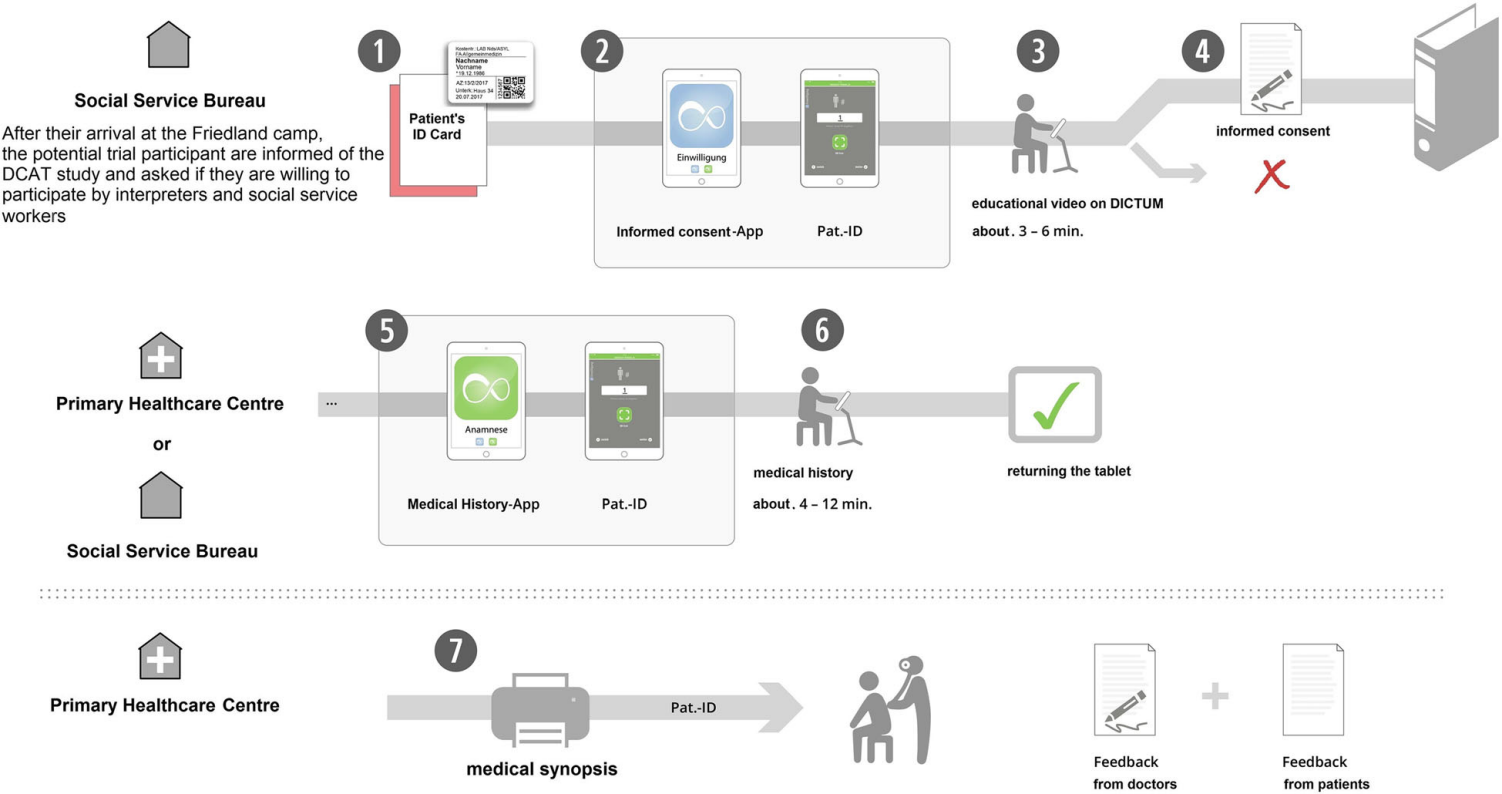
DCAT study procedures and implementation process

The potential participants are informed of the DCAT study directly on their arrival in Friedland camp through interpreters and social service workers. There are also posters and flyers (available in different languages) describing DCAT distributed across various sites in Friedland. Due to the structure of the referral system in Friedland, which mandates that asylum seekers need to get a permit from the responsible social service worker before being referred to the primary healthcare centre, we also included the social service bureau as a recruiting location in this study (see Fig. 4).

### Sample size calculation

The sample size calculation is based on the average numbers of patients in the last 12 months in Friedland. Due to fluctuations in refugee flows (ranging from 285 to 636/month), it is difficult to predict how many patients will be treated at the primary healthcare centre during the study period and meet the inclusion criteria, especially regarding the available languages. Despite these uncertainties, during the six months recruitment period we aim to include a total sample size of 880 participants (study group: 440 patients using DCAT, 440 patients not using DCAT). The sample size calculation is based on the consultation rates for medical treatment at the primary healthcare centre in Friedland as this is the primary outcome of the study protocol. Our previous research in the preparation phase revealed an average consultation rate for medical treatment of 2.65 (SD: 3.44) consultations per patient. Assuming a 10% dropout rate, to measure a minimal difference of 0.65 consultations per patient, 5% significance (two-sided) and a standard deviation of 3.5, 20 patients should be recruited per week to use DCAT to achieve the identified sample target.

### Study measurements

#### Quantitative measurements

- **Mode and frequency of consultation**

Every presentation of patients in the primary healthcare centre is registered through a coding system that depicts the time of the consultation as well as the condition presenting (e.g., medical treatment during consultation hours, medical emergency). Re-consultations of patients in the primary healthcare centre will also be recorded.

- **Reasons for Consultations and Diagnosis**

DCAT encodes automatically the estimated consultation reason as ICPC-2 code based on a patient’s input data [26, 27]. Diagnoses are documented afterwards from the patient notes made by the primary healthcare doctors. These are regularly kept for documentation purposes in the primary healthcare centre in Friedland.

- **Age, gender, asylum status, and affiliation to a language group**

Initially, patients using DCAT select their native language or dialect. This selection then directs further audio and visual information. Patient age, nationality, asylum status, sex are also extracted from the patient’s note.

- **Acceptance, usability, quality of communication, accuracy of DCAT information, and presence of ad-hoc (lay) interpreters**

When a patient finishes entering his/her data on DCAT, a digital questionnaire follows to assess patients’ experiences with DCAT (see Additional file 1). This questionnaire consists of two parts, the first consists of four questions and is applied directly after using DCAT. These questions seek to evaluate the acceptance and usability of DCAT. The final question regards the educational level of the patients.

The second section of the patient questionnaire follows after the consultation. It has five questions, which focus on the impact of using DCAT in the consultation situation, especially the communication and mutual understanding between the healthcare providers and the patients.

The attending physicians at the Friedland primary healthcare centre are asked to give their feedback on each consultation also using a two-part questionnaire (see Additional file 2). The first section comprises three questions, which ask the doctors their opinions on the perceived quality of communication and mutual understanding throughout the consultation. Whether ad-hoc interpreters are present is also documented. The second set of questions follows only when a medical report (synopsis) is produced through DCAT (see Additional file 3) and asks whether this printed report influences the consultation. Also, any inconsistencies between the DCAT collected information and clinical impressions of the doctors are sought.

#### Qualitative measurements

Throughout each phase of the DCAT study, the experiences of both healthcare professionals and patients with the intervention will be collected to understand DCAT effects and any additional questions. The research team will perform open and partially standardised interviews with healthcare workers (primary healthcare doctors and nurses) and a group of selected patients in Friedland [28].

The researchers also will periodically act as non-participant observers when patients are using the tool. This type of observation has proved its merit in earlier studies regarding objectivity and careful analysis [29, 30]. Additional comments will be collected and discussed with social workers and healthcare personnel to assess any issues and to react accordingly. Data will be collected in the form of field notes.

### Data management

Every patient in the DCAT study will receive a unique digital readable label (QR-Code), which is placed on his/her identification card (ID) in order to pseudonymise patient’s data. The social service workers issue these IDs to all refugees and asylum seekers after their arrival to Friedland. DCAT is launched after scanning the QR-Code. Therefore, the printed report, which has the QR-Code, can only be reassigned back to the patient by medical staff at the primary healthcare centre. Thus in case a report is lost, no one can identify it, which safeguards the patient’s personal as well as medical data.

Personal information allowing patient identification (e.g. first and last name) is not collected by DCAT. The collected data is not shared with other authorities or third parties. All other information not described above, e.g. health-related information given by the patient through DCAT is deleted permanently. The pseudonymised patient notes are evaluated by the research team and coded according to diagnosis (ICD-10 codes) [22].

All quantitative and qualitative data is only accessible by the study team. The study team is familiar with data privacy regulations and committed to data protection principles. A data monitoring committee is not needed in this trial because of the relatively short study period and the minimal risks to patient safety using the DCAT in comparison to randomised controlled trials on drugs and biologics.

All collected data will be permanently deleted ten years after the study.

### Data analysis

The quantitative data will be analysed using SPSS (version 25.0) on the basis of descriptives (i.e., frequencies and means). Differences between patients of the study group and the control group will be tested for statistical significance. Furthermore, differences between patients using DCAT and their physicians regarding their opinions on the perceived quality of communication and mutual understanding throughout the consultation will be tested for significance.

The qualitative data (interviews with GPs, nurses and patients) will be audio-recorded. Two members of the team will code the interview transcriptions and analyse them by the summarising qualitative content analysis [31].

## Discussion

While previous research has focused mostly on investigating the efficacy of interpreters and translators’ services to overcome language barriers in healthcare settings [32, 33], only some studies have evaluated the application of digital interpretation tools consistently at a primary healthcare level and its effects on health outcomes [13, 34]. Therefore, our study aims to assess the new DCAT in an action-oriented approach with mixed methods design. It ensures not only the involvement of patients but also different healthcare professionals (GPs and nurses). By following this approach, we reduce the limitations of previous research that focuses on only one aspect (either the patients or the healthcare providers) [35]. In addition, this study will provide new insights into the possible future benefits of DCAT in a primary care setting.

Earlier, past studies have had the tendency to evaluate the use of interpreters as a gold standard for dealing with the communication gap, especially in communities with high migration background [26, 32]. However, some recent studies have pointed to the limitations and restrictions of using interpreters and translators on a wide scale [33, 34, 36]. One of the key issues is patient confidentiality, which might be at risk if a third party is involved during the medical consultation [33, 35]. One of the many advantages of DCAT is that no third person is needed, which ensures the privacy of the patient’s personal and medical information.

By integrating different research methods, we expect to obtain a deeper understanding of the healthcare provider and patient perceptions. Indeed, Day et al. showed that following this approach for assessing such applications has better outcomes in terms of clinical settings [37].

During times when there are insufficient numbers of professional interpreters available to meet high numbers of patients (e.g. the 2015 refugee crisis in Europe), DCAT may represent a solution [38–40]. The need to develop practical solutions to facilitate communication for this group of patients during healthcare encounters is increasing especially after the unprecedented number of refugees and asylum seekers globally [16, 41]. Bischoff et al. have shown that language barrier costs are higher for healthcare provision among refugees and asylum seekers [10].

It should be noted however that DCAT is neither able to make a diagnosis nor can it give medical advice. It is an aid for doctors to obtain the medical history (symptoms and signs) from the patient perspective without the need for interpreters and translators.

We do expect some possible challenges with our study design. As this digital communication assistance tool is newly developed and its adoption will likely change the daily routine at the medical consultation, we are expecting some reluctance to use it, especially on the part of the healthcare provider. Such potential difficulties may require additional DCAT revisions during the study to ensure the quantitative as well as qualitative research goals (extension of the initial 18 month study period, changing the locations, where the patient can use DCAT, changing routine, e.g. admission times).

We also anticipate that not all patients will utilise DCAT because of unfamiliarity with the technology involved or the unavailability of some spoken languages at the primary healthcare centre in Friedland. During the study period, we will find out about such possible limitations and obstacles that might prevent DCAT usability.

In conclusion, this study remains an essential step to assess the potential effectiveness of the DCAT tool in facilitating communication and removing language barriers to improve the quality of the healthcare encounter for patients who do not speak German.

## Additional files

Additional file 1: Patient’s questionnaire, a two part digital questionnaire to assess patients’ experiences with the DCAT. (PDF 406 kb)

Additional file 2: Physician’s questionnaire, a two-part paper based questionnaire to collect physician feedback after each medical consultation. (PDF 92 kb)

Additional file 3: Medical synopsis, an example of a printed summary report of the patient’s provided complaints after using the DCAT. (PDF 237 kb)

## Abbreviations

AsylBLG: Asylbewerberleistungsgesetz (Services for Asylum Seekers Act)
DCAT: Digital communication assistance tool
GPs: General practitioners
ID: Identification card
