# Supplementary material for: Implementing a digital communication assistance tool to collect the medical history of refugee patients: DICTUM Friedland - an action-oriented mixed methods study protocol
Source: BMC Health Serv Res. 2019 Feb 6;19:103. doi: 10.1186/s12913-019-3928-1 (PMC6366114; doi:10.1186/s12913-019-3928-1)
Supplement: Supplementary file 1 — Patient’s questionnaire, a two part digital questionnaire to assess patients’ experiences with the DCAT. (PDF 406 kb) [file 12913_2019_3928_MOESM1_ESM.pdf]

Fragen an Patienten

Tab. 1 Fragen an Patient*in direkt nach der Anamneseerhebung

**a)** Konnten Sie Ihre wesentlichen Beschwerden in dem Programm eingeben?<br>ja (1), teilweise (3), nein (2)
**b)** Konnten Sie das Programm auf dem Tablet gut bedienen?<br>ja (1), teilweise (3), nein (2)
**c)** Haben Sie das Programm für sich benutzt oder für jemand anderen?<br>- Ich habe das Programm für meine eigenen Beschwerden benutzt (1)<br>- Ich habe das Programm für ein Kind bedient (2)<br>- ich habe das Programm für Angehörige bedient (3)
**d)** Haben Sie eine Schule besucht? (1) ja (2) nein<br>**e)** Falls ja, wieviel Jahre? ____

Tab. 2 Fragen an Patient*in, wenn sie die Medikamente abholen kommen

**a)** Waren Sie als Patient*in, als Angehörige*r oder als Elternteil eines erkrankten Kindes in der medizinischen Sprechstunde?<br>- als Patient*in (1)<br>- als Elternteil (2)<br>- als Angehörige*r/Bekannte*r (3)
**b)** Haben Sie die Kommunikationshilfe auf dem Tablet-PC verwendet?<br>(1) ja (2) nein
**c)** Hat die Ärztin oder Arzt Ihr Anliegen verstanden?<br>voll und ganz (1), weitgehend (2), teilweise (3), kaum (4), gar nicht (5)
**d)** Konnte die Ärztin oder Arzt Ihnen das weitere Vorgehen verständlich erklären?<br>voll und ganz (1), weitgehend (2), teilweise (3), kaum (4), gar nicht (5)
(nur wenn „**ja**“ bei Frage **b**)<br>**e)** Wie denken Sie: hat das Programm das gegenseitige Verständnis beeinflusst?<br>deutlich verbessert (1) etwas verbessert (2) gar nicht beeinflusst (3) etwas verschlechtert (4) deutlich verschlechtert (5)

Patients’ Questions

Tab. 1 Patients’ questions directly after collecting the medical history

**a)** Could you give your main complaints through this program?<br>yes (1), partially (3), no (2)
**b)** Could you use the program on the Tablet easy and well?<br>yes (1), partially (3), no (2)
**c)** Did you use the program for yourself or on behalf of somebody else?<br>- I have used the program for my own complaints (1)<br>- I have used the program on behalf of a child (2)<br>- I have used the program on behalf of a relative (3)
**d)** Have you been previously to a school? (1) yes (2) no<br>**e)** In case yes, for how many years? ____

Tab. 2 Patients’ questions, when they come to pick up the medications

**a)** Have you been at the consultation as a patient, as a relative or as a parent on behalf of a child?<br>- as a patient (1)<br>- as a parent (2)<br>- as a relative / acquainted (3)
**b)** Did you use the communication assistance tool on the Tablet-PC?<br>(1) yes (2) no
**c)** Did the doctor understand your concern?<br>Fully (1), to a great extent (2), partially (3), rarely (4), not at all (5)
**d)** Could the doctor explain to you further procedures clearly?<br>Fully (1), to a great extent (2), partially (3), rarely (4), not at all (5)
(Just in case of **yes** by question **b**)<br>**e)** How do you think the program has influenced the mutual understanding?<br>notably improved (1) slightly improved (2) no influence at all (3) slightly worsened (4) notably worsened (5)
