# Supplementary material for: Implementing a digital communication assistance tool to collect the medical history of refugee patients: DICTUM Friedland - an action-oriented mixed methods study protocol
Source: BMC Health Serv Res. 2019 Feb 6;19:103. doi: 10.1186/s12913-019-3928-1 (PMC6366114; doi:10.1186/s12913-019-3928-1)
Supplement: Supplementary file 2 — Physician’s questionnaire, a two-part paper based questionnaire to collect physician feedback after each medical consultation. (PDF 92 kb) [file 12913_2019_3928_MOESM2_ESM.pdf]

ID Etikett

Meine Meinung zur gerade stattgefundenen Konsultation:

1. Haben Sie das Anliegen des Patienten verstanden?

voll und ganz ☐ ☐ ☐ ☐ ☐ gar nicht

2. Hat der Patient Ihre vorgebrachten Empfehlungen (z.B. zur Medikamenteneinnahme) verstanden?

voll und ganz ☐ ☐ ☐ ☐ ☐ gar nicht

3. War ein Laiendolmetscher anwesend? Wurde durch einen Angehörigen (Laiendolmetscher) übersetzt? (z.B. englisch, deutsch)

ja ☐ ☐ nein

(bitte bei jeder Konsultation ausfüllen)

Nur, falls eine ausgedruckte Synopse vorliegt:

4. Wie hat der Ausdruck die Konsultation beeinflusst?

verbessert ☐ ☐ ☐ ☐ ☐ verschlechtert

5. Widersprechen sich Ausdruck und Ihr klinischer Eindruck vom Patienten?

ja ☐ ☐ ☐ nein

Vielen Dank für Ihre Unterstützung!

DICTUM-Projekt
Dr. Evelyn Kleinert
Tel. 0551-39-65302 od. -22638
evelyn.kleinert@med.uni-goettingen.de

UNIVERSITÄTSMEDIZIN GÖTTINGEN UMG
ALLGEMEINMEDIZIN

ID Label

My opinion on the current held consultation :

1. Did you understand the concerns of the patients ?

fully ☐ ☐ ☐ ☐ ☐ not at all

2. Does the patient understand your recommendations (e.g: taking medications)?

fully ☐ ☐ ☐ ☐ ☐ not at all

3. Was there a lay interpreter present? Was the translation performed by a relative of the patient (lay interpreter) ? (e.g. English, German)

Yes ☐ ☐ No

(Please to be filled in at every consultation)

Only , if there is a printed synopsis (Report):

4. How did the printed report influence the consultation?

improved ☐ ☐ ☐ ☐ ☐ worsened

5. Is there a conflict between the printed report and your clinical impression of the patient ?

Yes ☐ ☐ ☐ No

Thank you very much for your support

DICTUM-Projekt
Dr. Evelyn Kleinert, DICTUM-Projekt
Tel. 0551-39-65302 od. -22638
evelyn.kleinert@med.uni-goettingen.de

UNIVERSITÄTSMEDIZIN GÖTTINGEN UMG
ALLGEMEINMEDIZIN
