# Supplementary material for: Implementing a digital communication assistance tool to collect the medical history of refugee patients: DICTUM Friedland - an action-oriented mixed methods study protocol
Source: BMC Health Serv Res. 2019 Feb 6;19:103. doi: 10.1186/s12913-019-3928-1 (PMC6366114; doi:10.1186/s12913-019-3928-1)
Supplement: Supplementary file 3 — Medical synopsis, an example of a printed summary report of the patient’s provided complaints after using the DCAT. (PDF 237 kb) [file 12913_2019_3928_MOESM3_ESM.pdf]

Patient ID

#1700651

Date and time of the consultation

01.01.2018 – 16:16

Main symptoms

Important notes; Please be attention

CAVE:
Schwangerschaft möglich

Name

Vorname

Geb.-datum

[Patientenetikett]

In this field, an etiquet with basic information on the patient will be added

1234567

❶ Brustschmerz — Chest pain
❷ Beinschmerz — Leg pain
❸ Herzklopfen — palpitations

Allergien:
Pollen
Tierhaare
Medikamente

Information on Allergy

Vegetative medical history & Risk Factors

Vegetative u. Risiko-Anamnese:
Angaben vorhanden zu
Fieber
Schlafstörungen
Gewichtsveränderg.
Nachtschweiß

♀ 37 J.
ca. 160 cm
ca. 60 – 70 kg

Subj. Beschwerdedruck: (7) /10

Subjective estimation of symptoms strength

Drugs
Alcohol
Smoking

Drogen
Alkohol
Rauchen

Basic Information: Age, height and weight.

Chosen language

Sprachwahl:
Englisch

❶ Brustschmerz/ Herzbeschwerden

detailed questions and answers of chest pain

Der Patient/ die Patientin gibt an, seit 2 Tagen Schmerzen zu haben. Eine ärztliche Untersuchung ist bislang nicht erfolgt. Die Schmerzen sind seit dem Beginn stärker geworden.

Wie empfinden Sie die Schmerzen?
andauernd/ **mit Herzstolpern**

Strahlt der Schmerz aus?
Rücken

In welcher Situation treten die Beschwerden auf?
(**andauernd**/ **atemabhängig**/

Wie stark sind die Schmerzen?
mittel

Wo? (s. Abbildung Lokalisierung)

Liegen zusätzliche Symptome vor?
Luftnot /**Unterschenkelschwellung**

Besteht eine Herzerkrankung?
nein

Hatten Sie schonmal einen Herzinfarkt?
nein

Hatten Sie bereits eine Herzkatheteruntersuchung?
noch nie

Gibt es ein auslösendes Ereignis?
Unfall

Haben Sie eine Zuckererkrankung/Diabetes mellitus?
nein

Liegt eine Fettstoffwechselstörung vor?/erhöhte Cholesterinwerte/Blutfette?
nein

Haben Sie Bluthochdruck?
nein

Sind Sie übergewichtig?
nein

❷ Beinschmerz

detailed questions on leg pain

Wie lange bestehen die Beschwerden?
1 Woche

Was trifft auf Ihren Schmerz zu?
anhaltend

Wieviel Meter können Sie ohne Beschwerden gehen?
uneingeschränkt

Wo? (s. Abbildung Lokalisierung)

Sind außerdem aufgetreten?
Unfall/ **Beinschwellung**/ **Luftnot** /
Herzrhythmus-Störungen

Haben Sie Probleme, das Wasser oder Stuhl zu halten?
nein

❸ Herzklopfen

detailed questions on palpitations

Seit wann haben Sie die Beschwerden?
1 Tag

Sind die Beschwerden neu aufgetreten?
ja

Was spüren Sie?
